# Emergency Medical Services Encounters for Firearm Injuries — 858 Counties, United States, January 2019–September 2023

**DOI:** 10.15585/mmwr.mm7324a3

**Published:** 2024-06-20

**Authors:** Adam Rowh, Marissa Zwald, Katherine Fowler, Shane Jack, Carlos Siordia, Josh Walters

**Affiliations:** ^1^Epidemic Intelligence Service, CDC; ^2^Division of Violence Prevention, National Center for Injury Prevention and Control, CDC; ^3^biospatial, Inc., Durham, North Carolina.

**Summary**

**What is already known about this topic?**

Firearm-related deaths and injuries have increased in recent years.

**What is added by this report?**

During January 2019–September 2023, rates of emergency medical services (EMS) encounters for firearm injury were highest among males, non-Hispanic Black or African American persons, and persons aged 15–24 years. Annual rates during 2020–2023 exceeded the 2019 rate. The most substantial rate increases occurred in more urban counties and counties with greater income inequality, higher unemployment, and those with more severe housing problems.

**What are the implications for public health practice?**

The unequal distribution of high rates and increases in firearm injury EMS encounters highlight the need for states and communities to develop and implement comprehensive firearm injury prevention strategies to address the economic, social, and physical conditions that contribute to the risk of violence.

## Abstract

Firearm-related deaths and injuries have increased in recent years. Comprehensive and timely information on firearm injuries and the communities and geographic locations most affected by firearm violence is crucial for guiding prevention activities. However, traditional surveillance systems for firearm injury, which are mostly based on hospital encounters and mortality-related data, often lack information on the location where the shooting occurred. This study examined annual and monthly rates of emergency medical services (EMS) encounters for firearm injury per 100,000 total EMS encounters during January 2019–September 2023 in 858 counties in 27 states, by patient characteristics and characteristics of the counties where the injuries occurred. Overall, annual rates of firearm injury EMS encounters per 100,000 total EMS encounters ranged from 222.7 in 2019 to 294.9 in 2020; rates remained above prepandemic levels through 2023. Rates were consistently higher among males than females. Rates stratified by race and ethnicity were highest among non-Hispanic Black or African American persons; rates stratified by age group were highest among persons aged 15–24 years. The greatest percentage increases in annual rates occurred in urban counties and in counties with higher prevalence of severe housing problems, higher income inequality ratios, and higher rates of unemployment. States and communities can use the timely and location-specific data in EMS records to develop and implement comprehensive firearm injury prevention strategies to address the economic, social, and physical conditions that contribute to the risk for violence, including improvements to physical environments, secure firearm storage, and strengthened social and economic supports.

## Introduction

Multiple studies have highlighted recent increases in firearm-related deaths and injuries. For example, the annual firearm homicide rate increased 44% (from 4.4 to 6.3 per 100,000 persons) during 2019–2021 and remained elevated (5.9 per 100,000 persons) in 2022, and the firearm suicide rate increased 11% (from 7.3 to 8.1 per 100,000 persons) during 2019–2022.* Compared with 2019, in 2020, 2021, and 2022 the mean weekly number of firearm injury emergency department (ED) visits were 37%, 36%, and 20% higher, respectively (*1*). Syndromic surveillance of firearm-related injuries assessed in EDs has provided timely monitoring of trends, especially during the COVID-19 pandemic (*1*,*2*). Prehospital services (i.e., emergency medical services [EMS]) data have complemented ED surveillance of other injuries and conditions, including opioid overdoses (*3*). However, use of EMS encounter data to understand trends in firearm injuries is currently limited. EMS encounter data can provide information on the geographic location where firearm injury incidents occur, information that is often unavailable in hospital-based or mortality-related data sources and which could allow more refined analyses of social determinants of health associated with firearm injuries (*4*–*6*). Further, EMS encounter data capture nonfatal firearm injuries in persons who refuse or do not seek hospital-based care. This report describes trends in the rates of firearm injuries by selected patient- and county-level characteristics using EMS encounter data during January 2019–September 2023.

## Methods

EMS data collected by biospatial, Inc.^†^ from 858 U.S. counties with consistently high data coverage^§^ in 27 states^¶^ during January 2019–September 2023 were analyzed by month and year. A syndrome definition identified firearm injury EMS encounters by querying coded elements and narrative details of EMS patient care reports.** Firearm injury EMS encounters were calculated as rates per 100,000 EMS encounters. Annual rates and stratified subgroup rates during 2020–2023 were compared to corresponding prepandemic rates from 2019. Subgroups analyzed included patient characteristics (age group, sex, and race and ethnicity^††^) and characteristics of the county where the incident occurred. County-level characteristics were analyzed by linking EMS incident location information with data from the County Health Rankings and Roadmaps,^§§^ including unemployment rate,^¶¶^ income inequality ratio,*** prevalence of severe housing problems,^†††^ and urbanicity.^§§§^ Annual and monthly rates were calculated, and rate ratios (RRs) with 95% CIs were reported for calendar years 2020–2023 and compared with rates for 2019.^¶¶¶^ Analyses were conducted using R (version 4.2.2; R Foundation). This activity was reviewed by CDC, deemed not research, and was conducted consistent with applicable federal law and CDC policy.****

## Results

### Annual and Monthly Firearm Injury EMS Encounter Rates

**FIGURE 1:**
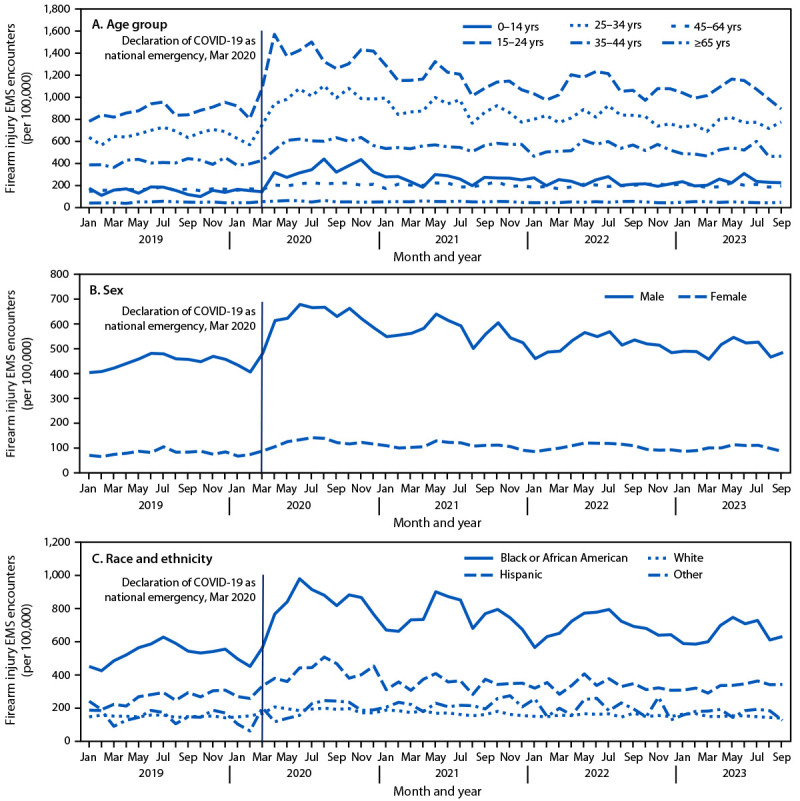
Monthly rate* of firearm injury–related emergency medical service encounters,^†^ by age group (A), sex (B), and race and ethnicity (C)^§^ — 858 U.S. counties, January 2019–September 2023 **Abbreviation:** EMS = emergency medical services. * Rates are reported per 100,000 total EMS encounters. ^†^ Encounters associated with firearm injuries were identified by querying dispatch information, chief complaint, narrative report, and diagnosis elements, according to a categorical syndrome definition based on the CDC Firearm Injury version 2 definition for Electronic Surveillance System for the Early Notification of Community-based Epidemics, which includes gunshot injuries sustained from handguns, rifles, and shotguns, and classification of injuries as unintentional, intentional self-harm, assault, legal intervention, terrorism, and undetermined intent. Injuries from air-powered, gas-powered, BB and pellet guns, and nonpenetrating injuries associated with firearms (e.g., “pistol whipping”) are excluded. ^§^ One race and ethnicity designation for each person was recorded. Persons of Hispanic or Latino (Hispanic) ethnicity, regardless of race, were classified as Hispanic. For the remaining categories, persons who were non-Hispanic are reported by their indicated single race classification (i.e., Black or African American or White). All persons of Asian, American Indian or Alaska Native, and Native Hawaiian or Pacific Islander classification were included in “Other” to overcome suppression of low cell counts. Persons with unknown or missing race or ethnicity were excluded.

**FIGURE 2:**
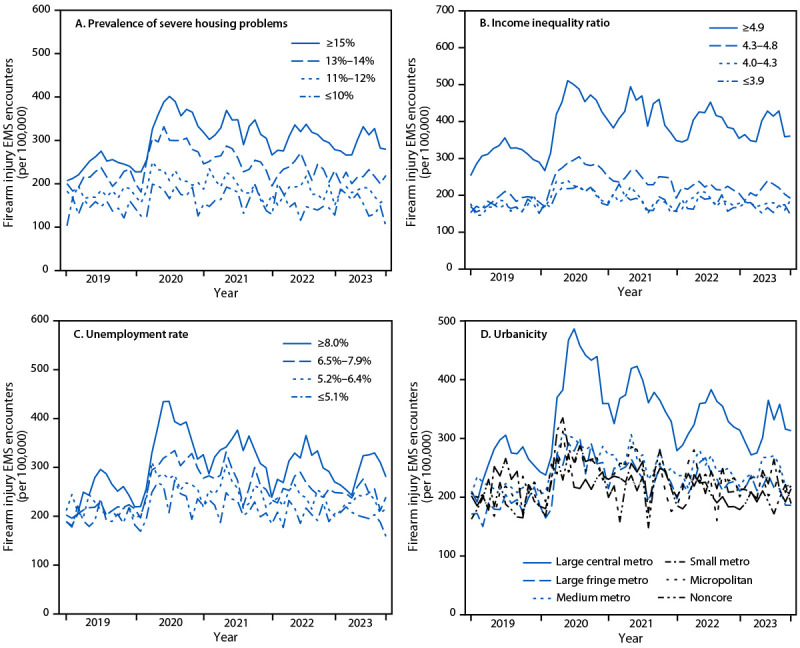
Monthly rate* of firearm injury–related emergency medical services encounters,^†^ by county-level prevalence of severe housing problems (A),^§^^,^^¶^ income inequality ratio (B),^§^^,^** unemployment rate (C),^§^^,^^††^ and urbanicity (D)^§^^,^^§§^ — 858 U.S. counties, January 2019–September 2023 **Abbreviation: **EMS = emergency medical services. * Rates are reported per 100,000 total EMS encounters. ^†^ Encounters associated with firearm injuries were identified by querying dispatch information, chief complaint, narrative report, and diagnosis elements, according to a categorical syndrome definition based on the CDC Firearm Injury version 2 definition for Electronic Surveillance System for the Early Notification of Community-based Epidemics, which includes gunshot injuries sustained from handguns, rifles, and shotguns, and classification of injuries as unintentional, intentional self-harm, assault, legal intervention, terrorism, and undetermined intent. Injuries from air-powered, gas-powered, BB and pellet guns, and nonpenetrating injuries associated with firearms (e.g., “pistol whipping”) are excluded. ^§^ Values were stratified into quartiles and analyzed using data from the County Health Rankings & Roadmaps 2023, University of Wisconsin Population Health Institute. http://www.countyhealthrankings.org ^¶^ Reported as the percentage of households experiencing severe housing problems. A household is defined as experiencing severe housing problems if the residence lacks functional plumbing or functional kitchen facilities, has overcrowding, or costs >50% of the household’s income. ** Defined as the ratio of county income at the 80th percentile to that at the 20th percentile. Upper quartile represents the greatest inequality. ^††^ Percentage of population aged ≥16 years who were unemployed but seeking work. ^§§^ Urbanicity analyzed according to the six strata specified by the National Center for Health Statistics Urban-Rural Classification Scheme for Counties. https://www.cdc.gov/nchs/data_access/urban_rural.htm

Compared with the annual firearm injury EMS encounter rate in 2019 (222.7 per 100,000 EMS encounters), the rate in 2020 was 32% higher (294.9), in 2021 was 27% higher (283.4), in 2022 was 17% higher (261.4), and in 2023 was 14% higher (252.8) (Table). After the declaration of COVID-19 as a national emergency in March 2020, a sharp increase in the monthly rate of firearm injury EMS encounters occurred among multiple demographic groups (Figure 1). An increase in the monthly rate of firearm injury EMS encounters was also observed across all levels of the county-level factors studied; increases were most pronounced in large central metropolitan counties and counties with a high prevalence of severe housing problems, high income inequality, and high unemployment (Figure 2). The total number of monthly EMS encounters decreased briefly during April and May 2020 before returning to prepandemic levels in June.^††††^

**TABLE:** Annual rate of firearm injury–related emergency medical service encounters* per 100,000 emergency medical service encounters, by patient- and county-level characteristics — 858 U.S. counties, January 2019–September 2023

Characteristic	2019	2020		2021		2022		2023	
	Rate	Rate	RR (95% CI)^†^	Rate	RR (95% CI)^†^	Rate	RR (95% CI)^†^	Rate^§^	RR (95% CI)^¶^
Total firearm injury EMS encounters**	**222.7**	**294.9**	**1.32 (1.30–1.35)**	**283.4**	**1.27 (1.25–1.29)**	**261.4**	**1.17 (1.15–1.19)**	**252.8**	**1.14 (1.12–1.16)**
**Patient-level characteristics**									
**Age group, yrs**									
0–14	148.5	290.6	1.96 (1.77–2.16)	256.8	1.73 (1.57–1.91)	226.2	1.52 (1.38–1.68)	235.0	1.52 (1.36–1.70)
15–24	875.7	1,277.7	1.46 (1.42–1.50)	1,161.6	1.33 (1.29–1.37)	1,094.2	1.25 (1.21–1.29)	1,045.0	1.21 (1.17–1.26)
25–34	667.5	931.1	1.39 (1.35–1.44)	890.1	1.33 (1.29–1.38)	822.6	1.23 (1.19–1.27)	758.0	1.15 (1.11–1.19)
35–44	413.0	552.3	1.34 (1.28–1.39)	552.9	1.34 (1.29–1.39)	543.0	1.31 (1.26–1.37)	507.7	1.24 (1.19–1.30)
45–64	164.0	200.8	1.22 (1.18–1.27)	203.8	1.24 (1.19–1.29)	197.7	1.21 (1.16–1.25)	201.4	1.23 (1.17–1.29)
≥65	48.0	53.8	1.12 (1.05–1.19)	54.1	1.13 (1.06–1.20)	49.2	1.03 (0.97–1.09)	49.1	1.02 (0.95–1.10)
**Sex**									
Female	81.7	112.5	1.38 (1.32–1.43)	110.0	1.35 (1.29–1.40)	104.3	1.28 (1.23–1.33)	99.7	1.22 (1.17–1.28)
Male	449.3	589.4	1.31 (1.29–1.34)	568.4	1.27 (1.24–1.29)	518.9	1.15 (1.13–1.18)	500.0	1.12 (1.10–1.14)
**Race and ethnicity^††^**									
Black or African American	537.0	770.4	1.43 (1.40–1.47)	758.5	1.41 (1.38–1.45)	692.4	1.29 (1.26–1.32)	656.3	1.23 (1.19–1.26)
White	151.0	181.7	1.20 (1.17–1.24)	170.1	1.13 (1.09–1.16)	156.6	1.04 (1.01–1.07)	152.2	1.00 (0.97–1.04)
Hispanic or Latino	262.1	393.8	1.50 (1.41–1.60)	348.1	1.33 (1.25–1.42)	336.6	1.28 (1.21–1.37)	332.5	1.32 (1.23–1.42)
Other	153.4	179.4	1.17 (0.98–1.40)	220.6	1.44 (1.22–1.70)	202.9	1.32 (1.12–1.56)	171.8	1.15 (0.94–1.41)
**County-level characteristics^§§^**									
**Prevalence of severe housing problems, %^¶¶^**									
≤10	145.1	166.0	1.14 (1.04–1.26)	164.9	1.14 (1.03–1.25)	147.2	1.01 (0.92–1.12)	151.7	1.04 (0.93–1.16)
11–12	178.3	205.9	1.15 (1.10–1.21)	193.8	1.09 (1.03–1.14)	179.7	1.01 (0.96–1.06)	179.0	1.01 (0.96–1.07)
13–14	213.0	277.4	1.30 (1.26–1.35)	253.6	1.19 (1.15–1.23)	230.9	1.08 (1.05–1.12)	214.3	1.02 (0.98–1.07)
≥15	242.7	333.4	1.37 (1.35–1.40)	326.3	1.34 (1.32–1.37)	302.7	1.25 (1.22–1.27)	293.9	1.21 (1.19–1.24)
Income inequality ratio***									
≤3.9	170.0	200.3	1.18 (1.11–1.25)	184.3	1.08 (1.03–1.14)	176.5	1.04 (0.98–1.10)	165.6	0.98 (0.92–1.05)
4.0–4.3	169.5	207.7	1.22 (1.18–1.27)	185.8	1.10 (1.06–1.14)	183.3	1.08 (1.04–1.12)	175.6	1.05 (1.01–1.10)
4.4–4.8	185.2	259.2	1.40 (1.36–1.44)	246.4	1.33 (1.29–1.37)	218.4	1.18 (1.14–1.22)	210.5	1.16 (1.11–1.20)
≥4.9	313.9	421.8	1.34 (1.31–1.38)	425.5	1.36 (1.32–1.39)	392.2	1.25 (1.22–1.28)	383.7	1.22 (1.19–1.25)
**Unemployment rate, %^†††^**									
5.1	198.1	226.9	1.15 (1.08–1.22)	218.6	1.10 (1.04–1.17)	203.1	1.03 (0.96–1.09)	197.6	1.01 (0.94–1.08)
5.2–6.4	216.2	258.8	1.20 (1.15–1.24)	248.9	1.15 (1.11–1.19)	234.4	1.08 (1.04–1.12)	227.8	1.04 (1.00–1.09)
6.5–7.9	210.9	289.7	1.37 (1.34–1.41)	276.1	1.31 (1.28–1.34)	254.8	1.21 (1.18–1.24)	246.9	1.19 (1.15–1.23)
≥8.0	248.9	343.6	1.38 (1.34–1.42)	333.2	1.34 (1.30–1.37)	303.1	1.22 (1.18–1.25)	291.7	1.18 (1.14–1.21)
**Urbanicity^¶¶¶^**									
Large central metro	261.2	382.3	1.46 (1.43–1.50)	371.2	1.42 (1.39–1.46)	332.7	1.27 (1.24–1.31)	314.7	1.22 (1.18–1.25)
Large fringe metro	185.5	247.9	1.34 (1.28–1.39)	235.9	1.27 (1.22–1.32)	227.0	1.22 (1.18–1.27)	213.5	1.18 (1.12–1.23)
Medium metro	218.2	271.0	1.24 (1.20–1.28)	262.1	1.20 (1.16–1.24)	245.7	1.13 (1.09–1.16)	244.4	1.12 (1.08–1.17)
Small metro	215.4	255.8	1.19 (1.13–1.25)	236.1	1.10 (1.04–1.15)	222.0	1.03 (0.98–1.08)	218.4	0.98 (0.93–1.04)
Micropolitan	205.0	250.3	1.22 (1.15–1.30)	243.4	1.19 (1.12–1.26)	223.9	1.09 (1.03–1.16)	223.0	1.12 (1.04–1.20)
Noncore	191.9	224.9	1.17 (1.09–1.26)	213.4	1.11 (1.04–1.19)	198.9	1.04 (0.97–1.11)	197.2	1.04 (0.96–1.12)

**Abbreviations:** EMS = emergency medical services; RR = rate ratio.

* Encounters associated with firearm injuries were identified by querying dispatch information, chief complaint, narrative report, and diagnosis elements, according to a categorical syndrome definition based on the CDC Firearm Injury version 2 definition for Electronic Surveillance System for the Early Notification of Community-based Epidemics (https://www.cdc.gov/nssp/php/onboarding-toolkits/essence.html), which includes gunshot injuries sustained from handguns, rifles, and shotguns, and classification of injuries as unintentional, intentional self-harm, assault, legal intervention, terrorism, and undetermined intent. Injuries from air-powered, gas-powered, BB and pellet guns, and nonpenetrating injuries associated with firearms (e.g., “pistol whipping”) are excluded.

**^†^** RRs reported for 2020–2023 are calculated with respect to the rate in 2019. An RR of 1 means that the rates were identical; an RR >1 reflects a rate higher than the rate in 2019.

^§^ The rate reported for 2023 reflects encounters during January–September 2023.

^¶^ RR reported for 2023 is calculated with respect to January–September 2019.

** Reported per 100,000 EMS encounters.

^††^ One racial and ethnic designation for each person was recorded. Persons of Hispanic or Latino (Hispanic) ethnicity, regardless of race, were classified as Hispanic. For the remaining categories, persons who were non-Hispanic are reported by their indicated single race classification (i.e., Black or African American or White). All persons of Asian, American Indian or Alaska Native, and Native Hawaiian or Pacific Islander classification were included in “Other” to overcome suppression of low cell counts. Persons with unknown or missing race or ethnicity were excluded.

^§§^ Values were stratified into quartiles and analyzed using data from the County Health Rankings & Roadmaps 2023, University of Wisconsin Population Health Institute. http://www.countyhealthrankings.org

^¶¶^ Reported as the percentage of households experiencing severe housing problems. A household is defined as experiencing severe housing problems if the residence lacks functional plumbing or functional kitchen facilities, has overcrowding, or costs >50% of the household’s income. Values were stratified into quartiles and analyzed using data from the County Health Rankings & Roadmaps 2023, University of Wisconsin Population Health Institute.

*** Defined as the ratio of county income at the 80th percentile to that at the 20th percentile. Upper quartile represents the greatest inequality.

^†††^ Percentage of population aged ≥16 years who were unemployed but seeking work.

^¶¶¶^ Urbanicity analyzed according to the six strata specified by the National Center for Health Statistics Urban-Rural Classification Scheme for Counties. https://www.cdc.gov/nchs/data_access/urban_rural.htm

### Firearm Injury EMS Encounter Rates by Patient Characteristics

By age group, annual rates of firearm injury EMS encounters were consistently highest among persons aged 15–24 years. The largest age group–specific increases in annual rates compared with rates in 2019 occurred among children and adolescents aged 0–14 years (Table). Annual rates were higher among males than among females (Table), but rate increases compared with rates in 2019 were larger among females. By race and ethnicity, the highest rates were observed among non-Hispanic Black or African American (Black) persons throughout the study period. Across all racial and ethnic groups and all study years, the largest single annual rate increase occurred among Hispanic or Latino (Hispanic) persons from 2019 to 2020. Annual rates among Black and Hispanic persons remained elevated through 2023; by 2023 rates in other racial and ethnic groups returned to prepandemic levels.

### Firearm Injury EMS Encounter Rates by County Characteristics

Annual rates of firearm injury EMS encounters were consistently highest during the study period in counties where severe housing problems were more prevalent (Table). Further, counties in the upper quartile of prevalence of severe housing problems experienced the most substantial increases in annual rates of firearm injury EMS encounters compared with rates in 2019. Similarly, annual firearm injury EMS encounter rates throughout the study period were highest in counties with the most income inequality, and rate increases compared with rates in 2019 were highest in counties in the upper quartiles of income inequality. Annual rates of firearm injury EMS encounters were highest in counties with higher unemployment rates; counties with the highest unemployment rates experienced the largest rate increases compared with rates in 2019. By urbanicity, annual rates and rate increases were highest in large central metro counties during 2020–2023 compared with 2019.

## Discussion

This study highlights the unequal distribution of firearm injury EMS encounters by individual- and county-level characteristics. At the onset of the COVID-19 pandemic, the rate of firearm injury EMS encounters increased overall and across most patient- and county-level characteristics, a trend observed elsewhere in prehospital data for penetrating trauma (*7*) and ED data on firearm injury (*1*). Overall and in most subgroups, annual rates of firearm injury EMS encounters remained higher during 2020–2023 compared with 2019; by 2023, however, rates generally decreased from their 2020 peak. The subgroup with the largest persistent elevation in 2023 was children and adolescents aged 0–14 years. Potential explanations for increased firearm injury rates during the COVID-19 pandemic and associated mitigation measures (e.g., stay-at-home orders) have been cited elsewhere and include increased firearm purchasing; changes in intimate partner violence patterns; changes in social support systems; and disruptions in health (e.g., limited access to mental health services), social, and emergency services (*8*,*9*).

The highest firearm injury EMS encounter rates occurred among persons aged 15–24 years, males, and Black persons; these findings align with previous findings from ED data on firearm injury (*1*), EMS data on penetrating injuries (*7*), and data on firearm-related deaths.^§§§§^ The highest rates and most substantial annual rate increases of firearm injury EMS encounters were observed in more urban counties and among counties with the highest prevalence of severe housing problems, largest income inequality, and highest rates of unemployment. These findings are consistent with a recent study using ED data on firearm injury from 10 U.S. jurisdictions, which found that rates of firearm injury ED visits were highest in communities facing greater social and economic disadvantages (*2*).

EMS encounter data, which can provide detailed information on the location of a firearm injury incident not typically available from ED visit data, could be paired with other data sources to help states and communities implement a comprehensive approach to firearm injury prevention, including strategies that promote financial security, economic opportunities, safe and stable housing, and resilient community infrastructure, and to evaluate the effects of prevention measures on firearm injuries over time (*10*). Future research linking injury location information from EMS data and treatment facility or patient residence information from other data sources could help further contextualize place-based risk and protective factors of firearm injury, assess the continuum of care for firearm injuries, and monitor patient outcomes.

### Limitations

The findings in this report are subject to at least six limitations. First, the data are not nationally representative; therefore, findings cannot be generalized beyond the 858 studied counties. Second, changes in health care use behaviors during 2020 might complicate interpretation of firearm injury rates during this period. Total EMS encounters decreased briefly early in the COVID-19 pandemic, which might have inflated the rate of firearm injury EMS encounters during this time; however, EMS use patterns rapidly returned to prepandemic levels. Third, the case definition used in this study captures firearm injuries overall and does not differentiate by intent, limiting the ability to understand whether encounters involved assaults, unintentional injury or self-directed violence. Developing intent-specific case definitions could improve research, surveillance, prevention, and response measures. Fourth, data used for this analysis do not represent injuries that were immediately fatal and did not involve EMS evaluation. Fifth, although the underlying EMS encounter data used for this analysis provided more detailed injury location information, County Health Rankings and Roadmaps data are reported at the county level, which required aggregation of EMS encounter data to the county level. Research examining variation in firearm injury EMS encounters at more geographically detailed levels is needed. Finally, data quality and completeness vary by EMS provider, reporting agency, location, and period.

### Implications for Public Health Practice

The unequal distribution of high rates and increases in firearm injury EMS encounters highlight the need for states and communities to develop and implement comprehensive firearm injury prevention strategies. Such strategies could include addressing underlying disparities in housing and economic security, creating protective community environments, implementing hospital and community-based outreach and violence interruption programs, and promoting secure firearm storage.^¶¶¶¶^

## Footnotes

* https://wonder.cdc.gov (Accessed April 5, 2024).

† EMS data were collected by biospatial, Inc., which receives EMS data from 44 states (27 full coverage [biospatial receives all records that the state office receives] and 17 partial coverage [biospatial receives some of the data from sources other than the state office, such as through partnerships directly with EMS providers]). Approximately 70% of EMS encounter data are available to data users within 24 hours (median = 11.1 hours). https://www.biospatial.io/

§ Underlying event coverage is a ratio of the records received by biospatial, Inc. compared with the estimated number of all EMS encounters expected for the specified geographic area (e.g., county); this metric is calculated using probabilistic models of historic data and county population characteristics. For this analysis, records from counties with underlying event coverage ≥75% for each quarter during the study period were eligible for inclusion.

¶ The following states that share data with biospatial, Inc. included at least one county that met the sufficient underlying event coverage threshold during the study period and were included in the analysis: Alabama (25/67 counties in the state), Alaska (7/29), Arizona (1/15), Arkansas (52/75), California (2/58), Colorado (35/64), Florida (42/67), Georgia (130/159), Idaho (3/44), Illinois (60/102), Kansas (75/105), Kentucky (90/120), Maine (16/16), Michigan (59/83), Mississippi (6/82), Montana (15/56), New Mexico (27/33), Oregon (1/36), Rhode Island (5/5), South Carolina (42/46), Texas (4/254), Utah (15/29), Virginia (76/133), Washington (1/39), Wisconsin (50/72), and Wyoming (18/23).

** Firearm injury EMS encounters were identified by querying dispatch information, chief complaint, narrative report, and diagnosis elements for gunshot injuries sustained from handguns, rifles, and shotguns classified as unintentional, intentional self-harm, assault, legal intervention, terrorism, and undetermined intent. Injuries from air-powered, gas-powered, BB and pellet guns; nonpenetrating injuries associated with firearms (e.g., “pistol whipping”); and aftercare for a firearm injury were excluded. Coded data elements were queried for relevant *International Classification of Diseases*, *Ninth Revision*, *Clinical Modification* diagnosis codes (including E965.0, E985.0, and E955.0) and *International Classification of Diseases, Tenth Revision* diagnosis codes (including W32–W33, W34.00, W34.09, W34.10, W34.19, X72, X73, X74.8, X74.9, X93, X94, X95.8, X95.9, Y22, Y23, Y24.8, Y24.9, Y35.01, Y35.02, Y35.09, and Y38.4).

†† One racial and ethnic designation for each person was recorded. Persons of Hispanic or Latino (Hispanic) ethnicity, regardless of race, were classified as Hispanic. For the remaining categories, persons who were non-Hispanic are reported by their indicated single race classification (i.e., Black or African American or White). All persons of Asian, American Indian or Alaska Native, and Native Hawaiian or Pacific Islander classification were included in “Other” to overcome suppression of low cell counts. Persons with unknown or missing race or ethnicity were excluded.

§§ Values were stratified into quartiles and analyzed using data from the County Health Rankings & Roadmaps 2023, University of Wisconsin Population Health Institute. http://www.countyhealthrankings.org

¶¶ Percentage of population aged ≥16 years who were unemployed but seeking work.

*** Defined as the ratio of county income at the 80th percentile to that at the 20th percentile. Upper quartile represents the greatest inequality.

§§§ Urbanicity analyzed according to the six strata specified by the National Center for Health Statistics Urban-Rural Classification Scheme for Counties. https://www.cdc.gov/nchs/data_access/urban_rural.htm

¶¶¶ For all purposes throughout the study, data from January–September 2023 were compared with data from the same period in 2019.

**** 45 C.F.R. part 46.102(l)(2), 21 C.F.R. part 56; 42 U.S.C. Sect. 241(d); 5 U.S.C. Sect. 552a; 44 U.S.C. Sect. 3501 et seq.

†††† A total of 11,606,344 EMS incidents occurred in the included jurisdictions in 2019; 11,580,929 in 2020 (99.8% of the 2019 total); 12,821,365 in 2021 (110.5% of 2019); 13,101,173 in 2022 (112.9% of 2019); and 9,778,920 in 2023 (114.6% of 2019, annualized for January–September). Monthly EMS volumes were 16.5% lower in April 2020 than in April 2019, and 10.4% lower in May 2020 than in May 2019.

§§§§ https://www.cdc.gov/injury/wisqars (Accessed April 5, 2024).

¶¶¶¶
https://www.cdc.gov/violence-prevention/php/resources-for-action/index.html

## References

[1] Zwald ML, Van Dyke ME, Chen MS, Emergency department visits for firearm injuries before and during the COVID-19 pandemic—United States, January 2019–December 2022. MMWR Morb Mortal Wkly Rep 2023;72:333–7. 10.15585/mmwr.mm7213a2 36995967 PMC10078842

[2] Van Dyke ME, Chen MS, Sheppard M, County-level social vulnerability and emergency department visits for firearm injuries—10 U.S. jurisdictions, January 1, 2018–December 31, 2021. MMWR Morb Mortal Wkly Rep 2022;71:873–7. 10.15585/mmwr.mm7127a1 35797204 PMC9290382

[3] Casillas SM, Pickens CM, Stokes EK, Walters J, Vivolo-Kantor A. Patient-level and county-level trends in nonfatal opioid-involved overdose emergency medical services encounters—491 counties, United States, January 2018–March 2022. MMWR Morb Mortal Wkly Rep 2022;71:1073–80. 10.15585/mmwr.mm7134a1 36006833 PMC9422964

[4] Hsia RY, Dai M, Wei R, Sabbagh S, Mann NC. Geographic discordance between patient residence and incident location in emergency medical services responses. Ann Emerg Med 2017;69:44–51.e3. 10.1016/j.annemergmed.2016.05.025 27497673

[5] Newgard CD, Sanchez BJ, Bulger EM, ; ROC Investigators. A geospatial analysis of severe firearm injuries compared to other injury mechanisms: event characteristics, location, timing, and outcomes. Acad Emerg Med 2016;23:554–65. 10.1111/acem.12930 26836571

[6] Mills B, Hajat A, Rivara F, Nurius P, Matsueda R, Rowhani-Rahbar A. Firearm assault injuries by residence and injury occurrence location. Inj Prev 2019;25(Suppl 1):i12–5. 10.1136/injuryprev-2018-043129 30928914 PMC7526511

[7] Huebinger R, Chan HK, Reed J, Mann NC, Fisher B, Osborn L. National trends in prehospital penetrating trauma in 2020 and 2021. Am J Emerg Med 2023;72:183–7. 10.1016/j.ajem.2023.07.022 37544146

[8] Miller M, Zhang W, Azrael D. Firearm purchasing during the COVID-19 pandemic: results from the 2021 National Firearms Survey. Ann Intern Med 2022;175:219–25. 10.7326/M21-3423 34928699 PMC8697522

[9] Rosenfeld R, Abt T, Lopez E. Pandemic, social unrest, and crime in U.S. cities: August 2020 update. Washington, DC: Council on Criminal Justice; 2021. https://build.neoninspire.com/counciloncj/wp-content/uploads/sites/96/2021/07/DESIGNED_FINAL1.pdf

[10] Branas CC, Kondo MC, Murphy SM, South EC, Polsky D, MacDonald JM. Urban blight remediation as a cost-beneficial solution to firearm violence. Am J Public Health 2016;106:2158–64. 10.2105/AJPH.2016.303434 27736217 PMC5104992

